# Geometry and Surface Feature Evaluation in E-PBF Process Using In-Operando Electron Emission Signal

**DOI:** 10.3390/ma19112362

**Published:** 2026-06-02

**Authors:** Abdulaziz Alfaifi, Omer A. Alshammery, Toan D. Truong, Haojun You, Mohsen Taheri Andani

**Affiliations:** 1 Department of Mechanical Engineering, Texas A&M University, College Station, TX 77843, USA; abdulaziz65@tamu.edu (A.A.); omeralshammery18@tamu.edu (O.A.A.); toan.truong@tamu.edu (T.D.T.); haojunyou@tamu.edu (H.Y.); 2 Department of Materials Science and Engineering, Texas A&M University, College Station, TX 77843, USA

**Keywords:** electron beam powder bed fusion, E-PBF, additive manufacturing, in-operando process monitoring, geometry assessment, in situ process monitoring, electron optical (ELO)

## Abstract

Electron beam powder bed fusion (E-PBF) requires reliable in situ process monitoring, and electron emission signals offer a promising avenue for this purpose. Most prior studies have relied on dedicated beam scans performed before or after melting, leaving open the question of whether the signal acquired during the melt itself can directly indicate geometric and topographical features of the fabricated part. In this work, the in-operando electron emission signal was recorded during spot-melting of a Ti-6Al-4V spur gear and evaluated for its ability to reconstruct geometric features and surface topography, with optical microscopy and profilometry serving as ground truth. A melt-pool dilation correction was applied to compensate for the geometric expansion of individual melt spots. After correction, the in-operando reconstruction reached agreement deviation values below 2.2% across the tooth tips, tooth bases, and chord widths, which are comparable to or better than those obtained from post-melt ELO imaging. Comparison with profilometer height profiles confirmed strong correlation with surface topography (Pearson 0.67–0.87 across all four profiles, *p* < 0.05 for all), indicating that the signal captures meaningful surface-topography variation in addition to geometric boundaries. The results demonstrate that the in-operando electron emission signal shows strong potential for in situ geometric and topographical assessment of complex parts in E-PBF, supporting its future integration into closed-loop process monitoring.

## 1. Introduction

Electron beam powder bed fusion (E-PBF) is a metal additive manufacturing process in which a focused electron beam selectively melts powder layers under vacuum to fabricate near-net-shape metallic components with complex geometries [1,2]. Compared with laser-based powder bed fusion, E-PBF offers several process-specific advantages, including high beam power, rapid electromagnetic beam deflection, reduced oxidation due to vacuum operation, and elevated build temperatures that can reduce residual stress and distortion [2,3]. These characteristics make E-PBF attractive for high-performance materials and geometrically complex components. However, despite these advantages, consistent part quality remains difficult to guarantee because the process is governed by strongly coupled thermal, physical, and material-dependent phenomena occurring across the powder bed, melt pool, and solidified surface [3,4].

A key challenge in E-PBF is that the final part quality is highly sensitive to powder spreading, preheating, sintering, melting, and solidification conditions. During preheating, powder particles partially sinter to improve electrical conductivity and reduce powder charging, but the sintering state depends on powder morphology, temperature distribution, beam parameters, and local packing conditions [5,6]. During melting, the beam–material interaction controls the melt-pool geometry, heat flow, surface morphology, and solidification behavior [7]. Variations in these conditions can contribute to local defects, surface irregularities, dimensional deviation, porosity, cracking, and non-uniform consolidation. Therefore, monitoring the process only after fabrication is insufficient for understanding when and where quality deviations originate.

In situ monitoring has therefore become an important research direction for metal powder bed fusion. General reviews of metal AM monitoring have emphasized the need for layer-wise and process-resolved sensing to connect process signatures with defect formation and part quality [8,9]. For E-PBF specifically, monitoring is more challenging than in many optical systems because the build chamber operates under vacuum, the process involves high temperatures, intense thermal radiation, metal vapor, and limited optical access [3]. Conventional optical or thermal imaging methods can provide useful information, but their implementation in E-PBF is constrained by chamber geometry, metallization of viewing windows, emissivity variation, and the difficulty of observing the melt region directly [10]. These limitations have motivated the development of electron-signal-based monitoring methods that use signals already generated by the electron beam–material interaction [3].

Electron-based monitoring is particularly attractive in E-PBF because the same beam used for melting or imaging can generate measurable emitted-electron signals. These signals may include backscattered electrons, secondary electrons, and thermionic electrons, depending on beam power, surface condition, local temperature, and interaction volume [11,12,13]. The physical basis is closely related to scanning electron microscopy, where image contrast arises from electron–matter interactions, surface tilt, atomic number effects, and detector geometry [11,12,13]. In E-PBF, these effects become more complex because the surface is not a polished static specimen; instead, it is a dynamic powder bed or molten surface with changing topography, temperature, and material state. As a result, the measured electron signal may contain information about both the local geometry and the process condition.

Electron optical imaging (ELO) has been widely investigated as an E-PBF monitoring approach. In ELO, the electron beam is raster-scanned over the powder bed or solidified surface, and emitted electrons are detected to reconstruct a spatial intensity map [14]. This enables layer-wise visualization of powder spreading, pre-melt conditions, and post-melt surface features without adding external optical hardware [14,15]. Prior studies have shown that ELO can support assessment of dimensional accuracy, surface condition, defect detection, and process quality evaluation [14,15,16,17]. More recent work has extended ELO toward surface topography reconstruction and build-surface characterization, demonstrating that electron optical signals can be related to surface height and local morphology when appropriate calibration or modeling is applied [18,19,20].

Despite these advances, ELO has important limitations. It is typically acquired before or after melting, meaning it represents a separate imaging step rather than the real-time melting event itself [14,15]. Pre-melting ELO can describe the powder bed condition, while post-melting ELO can describe the consolidated surface, but neither directly captures the signal generated during active melting. In addition, interpreting ELO contrast is non-trivial because the detected signal depends on surface tilt, detector position, beam incidence, electron yield, and material/topography effects [18,21]. Ray-tracing and electron-optical modeling studies have therefore been introduced to better explain how detector geometry and surface morphology influence the measured signal [18].

In parallel, in-operando electron-emission monitoring has emerged as a complementary strategy because it captures electron signals during the melting process itself. Arnold et al. demonstrated that backscattered-electron analysis during electron beam melting can provide process-relevant information from the active beam–material interaction [22]. Ledford et al. showed that real-time electron-emission monitoring can be applied to arbitrary geometries and toolpaths, indicating the potential of electron signals for spatially resolved process monitoring [23]. Recent work on in-melt electron analysis further suggests that signals acquired during melting can reveal process behavior that is not accessible from pre- or post-melting images alone [24]. These studies indicate that in-operando electron signals may contain information related to melt behavior, beam interaction, and local process stability.

Most existing in-operando and electron-based monitoring studies have focused on detecting process instabilities, cracks, inclusions, smoke events, or general anomaly signatures [17,25,26,27,28]. Machine-learning approaches have also been introduced for real-time anomaly detection using E-PBF monitoring data [27,28]. While these studies demonstrate the diagnostic value of electron-signal monitoring, the use of in-operando electron emission for direct geometric assessment remains less developed. In particular, limited work has examined whether the spatial distribution of signals acquired during melting can be used to evaluate the geometric accuracy and surface topography of the fabricated component itself, especially under spot-melting conditions rather than conventional line-scan interpretation.

The present work addresses this gap by assessing the hypothesis that the spatial distribution of in-operando electron emission signals, which are being acquired during spot-melting of Ti-6Al-4V, can directly indicate the geometric accuracy and surface topography of a fabricated component without requiring a separate post-melting imaging step. In comparison, ELO imaging requires a separate scan before or after melting and therefore adds process time and is limited to the consolidated surface state. The in-operando approach captures geometric information simultaneously with the melt and can in principle reflect both the geometric edges defined by scan strategy and the melt-pool driven surface features. Compared with post-process optical microscopy or profilometry, the in-operando signal provides this information directly inside the build chamber, removing the need for sample extraction and enabling integration into closed-loop process monitoring.

To evaluate this hypothesis, in-operando signal maps were compared with (i) electron optical images acquired before and after melting and (ii) ground-truth geometric and topographical measurements obtained using optical microscopy and profilometry. A dilation correction approach is introduced to compensate for the geometric expansion effects associated with the melt-pool size relative to the nominal beam diameter, and the unsampled region between every neighboring spot’s location, improving the agreement between signal-derived geometry and reference measurements. In addition to geometry evaluation, the study also examines the potential of in-operando signals to reveal surface topography features using a per-spot signal processing approach. Electron emission signals recorded at each melt spot are reconstructed as scatter maps in the X-Y plane and compared with surface morphology measurements obtained using profilometer. The comparison and correlation results showed strong agreement between the surface height map and the reconstructed signal. These observations suggest that in-operando electron signals can provide a direct indication of both geometric and topological characteristics without requiring extensive post-processing algorithms.

## 2. Materials and Experimental Methods

### 2.1. Complex Geometry Fabrication

To evaluate the in-operando ELO capabilities, a spur gear was fabricated. The geometry includes straight edges, fillets, and curved features of varying lengths, with up to 40 teeth and a pitch diameter of 38 mm, making it a geometrically complex object for measurement. The build was conducted on a freely programmable Freemelt ONE E-PBF system (Freemelt AB, Gothenburg, Sweden) using grade-23 Ti-6Al-4V powder (Tekna Plasma systems Inc., Sherbrooke, QC, Canada, 45–106 μm). Based on initial calibration, the focused electron beam diameter was about 250 μm, while the defocused beam diameter was roughly 1000 μm. All processing was conducted under vacuum pressure in the range of 10^−4^ to 10^−5^ mbar. These process parameters were optimized in-house for the Freemelt ONE platform and the present Ti-6Al-4V powder feedstock to provide stable spot-melting and reliable layer consolidation.

The object was consolidated on a 316L-stainless-steel substrate with a diameter of 100 mm and a thickness of 10 mm, positioned coaxially with both four BSE detectors(Freemelt AB, Gothenburg, Sweden) and the electron beam axis. Prior to fabrication, the substrate was leveled with the powder bed and located 160 mm below the BSE detectors (also known as working distance) along the Z-axis. Before melting, the substrate was preheated to approximately 650 °C by raster scanning the electron beam across its surface. This preheat temperature was maintained throughout the build and monitored using a type-N thermocouple.

Spot-melting, employing single-directional shifted strategies, was used to print the object. A focused electron beam operating at a power of 800 W and an area energy density (AED) of approximately 3.5 J/mm^2^ was employed. Each melt spot was positioned 0.25 mm away from its neighboring spot. For each layer, a complete processing cycle was executed, consisting of powder raking (Step 1), preheating (Step 2), pre-melting ELO imaging (Step 3), melting (Step 4), and post-melting ELO imaging (Step 5).

### 2.2. Electron-Optical (ELO) Imaging

The detection system in our machine consists of a multi-segment BSE detector positioned above the build plane. Those built-in four-quadrant diode configurations are capable of capturing directional variations in the emitted electron flux. The signals collected from the detector segments are amplified and digitized to produce intensity maps representing the interaction between the electron beam and the material surface [18].

ELO imaging is applied during two stages of the E-PBF process: pre-melting stage and post-melting stage [4,29]. In the pre-melting stage, the electron beam scans the freshly deposited powder before melting. This step enables visualization of powder bed characteristics, such as layer uniformity and particle distribution [29]. The BSE signal in the pre-melting stage is influenced by the complex interaction of the electrons with loosely packed powder particles [18]. The complexity is because of the multiple scattering events and variations in local powder height, which affect the probability of electron escape and thus the detected signal intensity [18]. Consequently, variations in signal intensity can provide information about powder layer morphology and spreading quality [29].

On the other hand, post-melting ELO imaging is performed to inspect the consolidated surface of the fabricated layer [15]. In this stage, the electron beam interacts with a solid metallic surface, where the backscattered electron yield is strongly dependent on both the effective atomic number of the material and the local surface orientation relative to the detector [15]. By comparing the intensity differences measured by the individual detector segments, it is possible to infer surface slope information [18]. Such capabilities allow the detection of surface defects, including cracks, pores, and irregular melt tracks, making ELO imaging a valuable tool for layer-wise quality monitoring and defect identification in E-PBF processes. Typical scanning parameters for these ELO captures in our experiment are shown in Table 1.

**Table 1:** ELO scan parameters used to capture images for pre- and post-melting stages.

Step	Process Parameters	
Pre- and post-melting	Spot size, d (mm)	0.250
	Hatch spacing, h (mm)	0.125
	Scan velocity, v (mm/s)	8000
	Power, P (W)	60
	Energy density, E (J/mm^2^)	0.06

### 2.3. In-Operando Spot-Melting Data Collection

In contrast with the previous scanning technique, in-operando data for spot-melt experiments, as conducted here, were not processed as images since this is not feasible due to the spatial resolution being incredibly low. This led to reduced image quality and limited defect-detection and geometry monitoring capabilities. The acquisition rate for the BSE detectors is 250 kHz, which will result in the acquisition of ~25 samples per 100 µs dwell time. To capture the full electron emission at each melt coordinate, these samples were summed for every spot. The purpose of summing this electron emission is to capture and gather all the emitted signals for the full beam–material interactions [30]. Furthermore, summing up the signal will give a correct representation of process parameters’ reflection on the signal captured during the melt, while averaging could present a misleading interpretation of the signal. These interactions can represent multiple events during the melt, such as BSE signal generation of the targeted material, thermionic electron generation, starting the melting stage, indication for the evaporation of material, and an indicator of the surface status. Also, in-melt signal can reflect the change in process parameters and could give an indication on the delivered energy density [24].

### 2.4. Geometry Accuracy Assessment

The main reference to evaluating the geometrical accuracy of the fabricated gear was the Olympus DSX 1000 Optical Microscope (OM) (Evident Scientific, Tokyo, Japan). It operates by illuminating a sample with controlled light and using a system of objective lenses and a digital camera to magnify and capture high-resolution images. The OM image captured the full gear geometry within a single field of view, which allows consistent boundary extraction across the entire circumference. The spatial calibration was performed using the microscope scale bar to convert pixel coordinates into physical units.

This calibration approach introduces a small systematic uncertainty associated with the finite pixel resolution and the precision of scale-bar endpoint identification. Direct measurement of the calibration image showed that the 5 mm scale bar spans 433 pixels, corresponding to a spatial calibration factor of 11.55 μm/pixel. Assuming a ±1 pixel uncertainty in scale-bar endpoint localization, the relative calibration uncertainty is therefore ±0.231% (1/433), which corresponds to approximately ±23 μm on a 10 mm feature or ±88 μm across the 38 mm pitch diameter. This calibration uncertainty is several times smaller than the agreement deviation values reported in Table 2 (which are on the order of 0.8–1.5% of the feature radius) and is therefore not a limiting factor in the comparison between OM-derived ground truth and the in-operando measurements.

Although this calibration approach may introduce a small systematic uncertainty associated with pixel resolution and scale bar measurements. The high spatial resolution of the optical imaging system ensures that uncertainty is negligible compared with the geometric deviations evaluated in this study. Taking OM image as a reference allows real ground truth comparison of the final fabricated part. The high-resolution OM image was then evaluated by comparing spatial information obtained from both ELO imaging and in-operando data. The objective of the comparison was to determine how accurate in-operando signal monitoring reflects the true geometry of the part compared to the more mature and previously tested ELO imaging. Because of the differences in acquisition methods, there will be differences in spatial resolution and coordinate representations. Therefore, to ensure an accurate approach, a unified approach was developed to enable consistent geometric evaluation.

**Figure 1:**
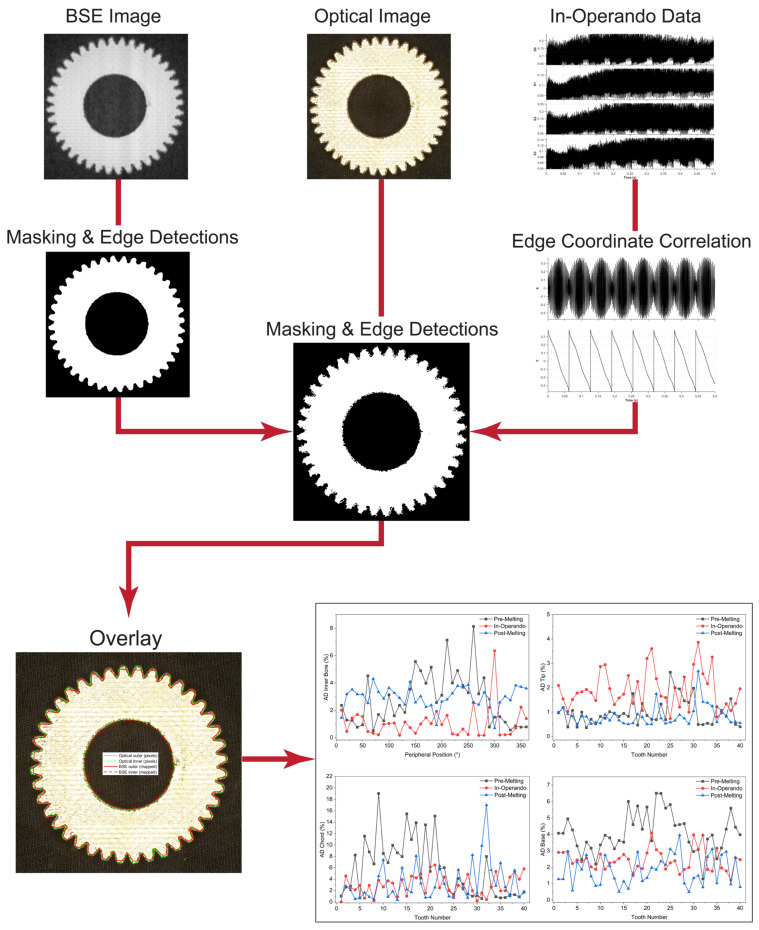
Schematic of the analysis workflow used in this study. The fabricated spur gear is characterized by three independent sources: (i) the in-operando electron emission signal acquired per spot during the melt; (ii) pre- and post-melt electron optical (ELO) images; and (iii) optical microscopy (OM), which serves as ground truth. Image-based and coordinate-based geometric data are processed in parallel, each yielding boundary representations of the part, which are then compared against the OM reference to quantify the agreement deviation at every measured feature.

The optical image of the fabricated component was first taken with intentionally high contrast since the target here is to extract the edges easily and to prevent any noise from disrupting the masking. The masking process starts by turning the image to a grayscale to enable automatic thresholding. After that, the image was calibrated using scale reference (known physical length). Then, to start turning the image to a known coordinate, a spatial calibration factor needed to be calculated using the physical length and the corresponding spanned pixels in the image:

(1) $$s=\frac{L_{mm}}{L_{px}}$$

where *L_mm_* denotes the scale bar physical length, *L_px_* represents the equivalent pixels spanned by the scale bar, and s denotes the spatial calibration factor. This will allow the conversion of pixel coordinates to physical units by using the calibration factor as follows:

(2) $$x\;=\left(x_{px}-{\;x}_{c}\right)\;s$$

(3) $$y=\left(y_{px}-{\;y}_{c}\right)s$$

where (*x_c_*, *y_c_*) represents the centroid of the extracted gear mask shown in Figure 1, and (*x*, *y*) are the new physical coordinates.

To ensure an accurate alignment between different images or any other compared source, the OM image was first taken with careful alignment with where it was located in the melt chamber. Since there will be geometry comparisons, polar coordinates will be much more convenient to locate and compute the agreement. Therefore, the radial distance r and the angular coordinates θ need to be calculated as follows:

(4) $$r\;=\sqrt{x^{2}\;+y^{2}}$$

where both *x* and *y* represent the Cartesian coordinates. The angular coordinate can be obtained from the following:

(5) $$\theta \;=\;\tan^{-1}\left(\frac{y}{x}\right)$$

This polar representation enables direct comparison between the two datasets at identical angular locations. Also, it helps to separate individual teeth along the circumference.

Even though the optical image was aligned carefully, there might be some kind of misalignment compared to ELO images. Therefore, a standard rotation matrix will be applied if necessary:

(6) $$R\left(∆\theta \right)=\left[\begin{array}{cc} \cos∆\theta & -\sin∆\theta \\ \sin∆\theta & \cos∆\theta \end{array}\right]$$

where ∆θ represents the angular difference of a chosen gear tooth of optical image boundary and any other reconstructed boundaries.

After ensuring the alignment, individual gear teeth were segmented by identifying local maxima in the radial profiles of the optical geometry boundaries. These maximum points correspond to tooth tips, while the minimum between adjacent maximum points is considered the teeth bases. This segmentation ensures each tooth is to be identified separately.

#### 2.4.1. Pre- and Post-Melting

**Figure 2:**
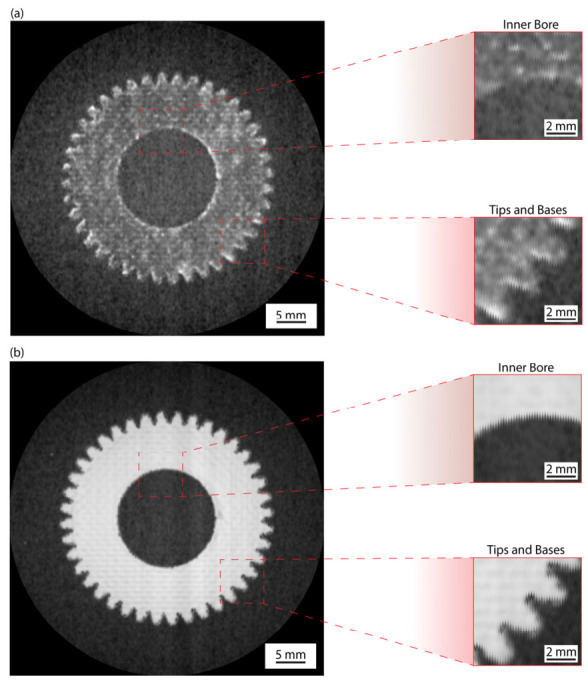
Example electron optical (ELO) images of the spur gear acquired (**a**) before and (**b**) after the spot-melting process. The pre-melt image (**a**) shows the powder bed prior to consolidation, with the gear footprint faintly visible against a high noise speckled background characteristic of loose powder. The post-melting image (**b**) shows the consolidated part with sharp contrast between the melted gear surface and the surrounding unfused powder. Insets on the right highlight two representative regions, which show the inner bore (in the top) and the tip and base region of the teeth (bottom). Those insets illustrate the significant increase in signal contrast and edge definition between pre- and post-melt acquisition.

For the pre- and post-melting stages, geometric information was extracted directly from BSE images acquired before and after the melting process. Each image provides a map of BSE intensities, which represent the powder bed and solidified geometry, respectively. Starting the processing by spatially calibrating the images using a scale bar. The calibration converts pixel coordinates to physical units through a calibration factor following Equation (1). The gear region of interest was then isolated via cropping, followed by binary mask generation. The binary mask was generated using intensity thresholding and morphological operations to remove background noise and improve feature boundaries. The mask centroid was used as the origin for coordinate alignment (Equations (2) and (3)) to ensure consistent positioning relative to the optical image reference. Gear teeth boundaries and central holes were detected by using edge tracing. Equations (4) and (5) demonstrate the formula used to transfer the Cartesian coordinates to polar coordinates to enable direct evaluation of radial distance at specific positions. Lastly, local maxima and minima in radial were used to segment individual teeth, while a smoothing filter suppressed high-frequency noise. This workflow was applied independently to both pre- and post-melting BSE images, allowing for radial deviation quantification related to reference geometry. Representing pre- and post-melting ELO images of the fabricated gear are shown in Figure 2, illustrating the contrast difference between loose powder and consolidated material.

#### 2.4.2. In-Operando Spatial Correlation

For the in-operando case, the geometric information was not extracted from a post-processed image, but directly from the raw multi-channel data acquired during the melting. The detector signal collected from the four BSE channels were recorded simultaneously with the beam position coordinates. This instantaneous location registration will allow the part geometry to be reconstructed in spatial form during the build rather than after or before the melting. These signals that have been collected from the four channels will first be combined with each other, since they share the same location, to represent the fully acquired emission from that specified coordinate.

(7) $$S_{BSE}=S_{1\;}+S_{2}+S_{3}+{\;S}_{4}$$

where *S*_1_, *S*_2_, *S*_3_, and *S*_4_ are the signals from the four BSE detectors. A short median filter was then applied to suppress isolated noise. The beam position coordinates that have registered were converted from arbitrary units to physical units by multiplying it to a scaling factor. This will allow the representation of the collected data onto a regular grid.

Unlike conventional image-based edge detection, the potential geometric edges were identified by the designed distance between adjacent melt spots. The pre-identified distance between spots in all directions is 250 μm. As a result, having a signal intensity with no neighboring signal in one or more directions will be identified as an edge point. Later those edge points will be connected to form a continuous boundary line to create the designed geometry edges. Having this distance between spot-melts will reduce the resolution of the collected data, unlike the post- and pre-melting scans, which are designed for constructing images. Therefore, further processing is needed to accurately measure the geometry, since even though the calibrated beam diameter is 250 μm, the melt pool will surely be much more than that. The processing method will be evaluated and discussed in the following few sections.

## 3. Results and Discussion

### 3.1. Geometry Agreement Deviation

After processing both image- and signal-based data and applying all the alignments and segmentations required, several geometric agreement metrics were computed to capture various aspects of gear geometry. The geometry extraction algorithm was executed five independent times for each dataset to evaluate the repeatability of the procedure. These repeated runs correspond to independent executions of the geometry-extraction and alignment workflow on the same dataset rather than repeated fabrication experiments. The agreement deviation values reported in this study were calculated from the averaged results of these runs, while the corresponding standard deviations were used to quantify the variability introduced by the processing algorithm. In addition, the geometric comparisons were performed across all 40 gear teeth, providing multiple spatial sampling points around the circumference of the fabricated component.

**Figure 3:**
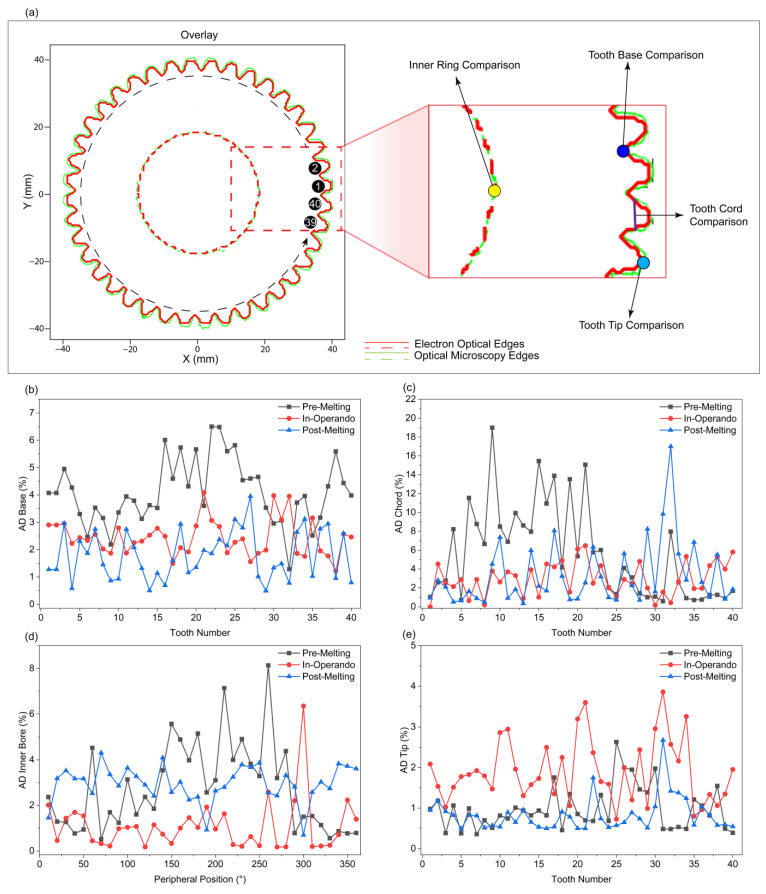
Geometric agreement between the in-operando, post-melt ELO, and OM measurements of the fabricated gear, evaluated before dilation correction. (**a**) Overlay of the geometric borders extracted from BSE and OM images, showing the per-tooth points used for the comparison. The teeth order in the comparison starts from 1 and ends at 40 going counterclockwise. (**b**) Per-tooth agreement deviation at the 40 tooth bases. (**c**) Per-tooth agreement deviation of the chord width across the 40 teeth. (**d**) Inner bore boundary sampled at 10° intervals clockwise from the starting point. (**e**) Per-tooth agreement deviation at the 40 tooth tips. Error bars in (**b**–**e**) represent the standard deviation across the independent measurement runs.

**Figure 4:**
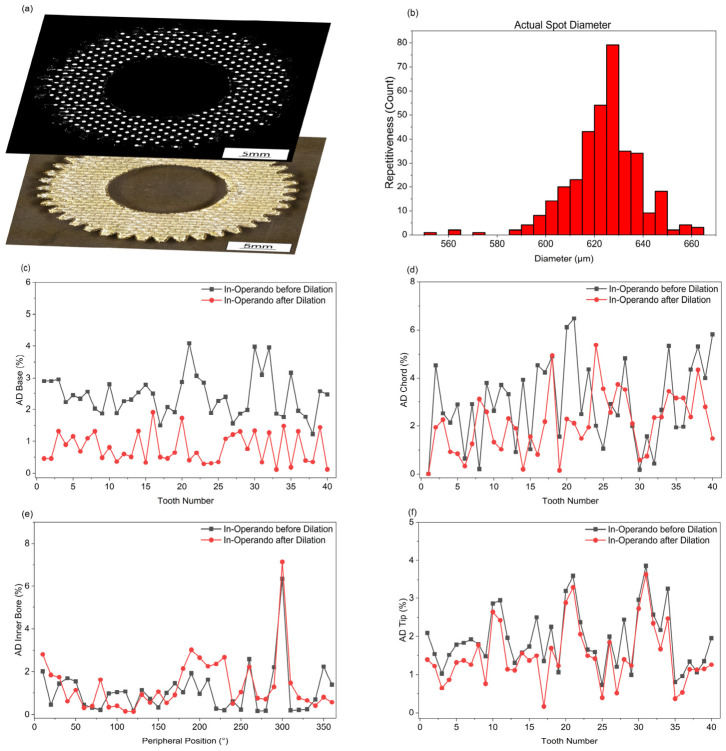
Melt-pool measurement and effect of the dilation correction on the in-operando geometric reconstruction. (**a**) Direct measurement of individual melt pools on the printed object surface from the post-melt OM image, used to obtain the mean melt-pool diameter. (**b**) Histogram of the measured melt-pool diameters (*n* = 356), yielding a mean diameter of 623 μm with a coefficient of variation of 2.4%. (**c**–**f**) Per-tooth (or per-position) agreement deviation between in-operando and OM-derived measurements before and after applying the dilation correction at (**c**) the tooth bases, (**d**) the tooth chord widths, (**e**) the inner bore (sampled at 10° intervals), and (**f**) the tooth tips.

Specifically, the agreement deviation values shown in Figure 3 and Figure 4 were computed per tooth using Equations (8)–(13) for each of the 40 teeth, and the radial position of the local extremum (tip and base) was extracted from each measurement source (in-operando, pre-melt ELO, post-melt ELO) using Equations (1)–(7). Then, it was compared against the corresponding position in the OM ground truth image. The chord width agreement deviation was likewise evaluated tooth by tooth at the position *β* = 0.5 between tip and base radii, and the inner bore deviation was sampled at 10° angular intervals around the bore boundary. The first metric evaluates the agreement at the tooth tips (Figure 3a), which represents the addendum region of the gear. The relative deviation at the *n-th* tooth tip is defined as follows:

(8) $$AD_{tip,n}\;=\frac{\left|r_{bse}\left(\theta_{n}\right)\;-\;r_{opt}(\theta_{n})\right|}{r_{opt}(\theta_{n})}\;\times 100$$

where $r_{opt}$ and $r_{bse}$ represent the optical and the BSE radii evaluated at the angular position of the tooth tip. Similarly, the agreement at the tooth base was evaluated using the following:

(9) $$AD_{base,n}\;=\frac{\left|r_{bse}\left(\theta_{n}\right)\;-{\;r}_{opt}(\theta_{n})\right|}{r_{opt}(\theta_{n})}\;\times 100$$

which captures the agreement at the dedendum region.

After calculating the main two points in gear tooth (tip and base), calculating any point between them on either the left or right tooth flank is possible. The following formula will be used to determine the radius of any point of comparison that falls between the base and the tip $r_{\beta }$:

(10) $$r_{\beta \;}={\;r}_{base}+\;\beta \;\left(r_{tip}\;-\;r_{base}\right)$$

where the *β* value can be chosen between 0 and 1, where 0 means the radius is exactly at the base and 1 exactly at the tip. In this comparison, *β* will be chosen at the halfway point between the tip and the base.

Since the radius at the desired point to calculate the tooth width is already found, the angle between the two points that intersect with the radius on both flanks is needed to find the chord length. This was made possible by previously turning the Cartesian coordinate system into a polar coordinate system, by subtracting the angle coordinate of the intersected points on both flanks, which are predefined to be 0.5 of flanks lengths. To calculate the chord with *C_opt_*_&_ *_ELO_* the following formula will be used:

(11) $$C_{opt\;\&\;ELO}=\;2r_{\beta }\sin\left(\frac{∆\theta }{2}\right)$$

where ∆θ is the angle difference between the intersection points.

However, the in-operando chord width is directly calculated from the distance between the intersection points:

(12) $$C_{in-operando\;}=\;\sqrt{{\left(x_{R}-{\;x}_{L}\right)}^{2}\;\;+\;\;{\left(y_{R}\;-\;y_{L}\right)}^{2}}$$

where the subscript for the coordinates in *x* and *y* refers to the intersection points with the right and left flanks of the gear teeth. Then the agreement deviation will be calculated as before to determine the tooth width agreement deviation.

Finally, the inner gear bore geometry was evaluated independently of the tooth features. The inner radius was sampled at regular angular intervals of 10°, beginning from the orientation of the first detected tooth. The deviation at angular location is defined as follows:

(13) $$AD_{bore,n}\;=\;\frac{\left|r_{bse}\left(\theta_{i}\right)\;\;-\;\;r_{opt}(\theta_{i})\right|}{r_{opt}(\theta_{i})}\;\times \;100$$

where $\theta_{i}$ refers to the inner bore at specified locations.

Throughout this approach, geometrical agreement between the OM and diverse types of BSE measurements was evaluated at multiple gear features (Figure 3).

In Figure 3b, the deviation at the tooth base reveals that the pre-melting exhibits the highest and the most erratic error, fluctuating between 1.5% and 7%. On the other hand, post-melting showed the lowest overall agreement deviation since it consistently drops below 4%, while the in-operando falls in between the post- and pre-melting AD. The high error during the pre-melting is a direct consequence of the particulate nature of the non-melted powder bed, where partially sintered powder in tight, concave roots obscures sharp edge detection. In contrast, once the material is melted and allowed to consolidate, the boundaries become a dense solid. The deviation stabilization confirms that the solidified material provides a much superior surface for edge detection compared to the rough powder layer topology.

Figure 3c focuses on the tooth chord width, where the pre-melting stage performs extremely poorly, showing deviation reaching up to 20%. The powder contour simply fails to accurately represent the thickness of each tooth. Post-melting showed remarkably high agreement, which provides the lowest baseline and the closest match to the OM reference. In-operando, on the other hand, showed very close agreement with the post-melting images, but still with a little bit more error. The powder bed offers only a loose approximation of the geometry since the contrast is not apparent as in the post-melting images. However, the post-melting measurement captures the full boundaries after the thermal expansion effect has occurred. Tracking the final state is essential for bulk features, which makes post-melting the most reliable indicator of the final chord dimension.

The inner bore deviation presented in Figure 3d demonstrates that the pre-melting is exposed to considerable fluctuation, reaching up to approximately 8%. In contrast, in-operando has the highest accurate indicator of the final geometry, holding the lowest deviation, even lower than the post-melting stage, yielding flatness and the most stable deviation profile. In the spot-melting strategy, continuous curves, like an inner bore, are challenging to fabricate since they require a highly optimized jump sequence and overlapping spots to ensure a smooth boundary [31]. Once the sequence is completed and the layer fully solidified, the post-melting ELO image and in-operando coordinate-driven data accurately capture the finalized, smooth structural boundaries, which explains its excellent geometric fidelity.

Figure 3e examines a unique trend in the geometry of the tooth tips. The pre-melting stage yields the lowest deviation, outperforming the post-melting stage. While pre-melting captures the boundary with the lowest overall deviation, under 3.5%, the post-melting shows a slightly higher deviation, approximately 4%. Generally, post-melting should provide the closest agreement with OM because it represents fully consolidated geometry, however, this is not the case here. One of the reasons could be the retention of heat expansion directly after the melting, which explains the minor difference shown in Table 2. Another thing is the localized discrepancy in spreading the powder at the tooth tips, which results in good contrast, allowing the location to be captured correctly with reduced thermal expansion.

### 3.2. Melt Spot Dilation

Before discussing the dilation correction, it is useful to characterize the inherent resolution limits of the in-operando method. The spatial resolution of the in-operando method is set by two physically measured quantities: the spot spacing of 250 μm, which establishes how often the signal is sampled across the surface, and the mean melt-pool diameter of 623 μm (measured directly from n = 356 melt pools), which determines the physical extent of each sampled region. Features smaller than the spacing between two adjacent samples cannot be distinguished from one another, while features smaller than the melt-pool diameter are physically averaged over the melt pool itself. The minimum detectable geometric feature size is therefore set by the larger of these two scales, approximately 600–700 μm. All features measured in this study (tooth tip, tooth base, chord width, and inner bore) have characteristic dimensions exceeding 1 mm and are therefore well above this resolution limit.

Local curvature can also contribute to measurement bias when the melt-pool diameter is significant relative to the feature radius. An empirical upper bound on this contribution is provided by the residual agreement deviation measured at each feature after dilation correction (Table 2): 1.36% at the inner bore, 1.51% at the tooth tips, 0.80% at the tooth bases, and 2.12% at the chord widths. These residuals encompass all uncorrected effects, which include any curvature-induced bias, and bound the curvature contribution at well below 2.5% of the feature scale in every case. The systematic trend that the tooth tips and chord widths (the features with the smallest local radii of curvature in the gear geometry) exhibit the largest residual deviations is consistent with this interpretation. The method is consequently expected to perform best for features substantially larger than the melt-pool diameter and with smooth, large-radius curvature; finer features or significantly tighter curvatures would require smaller spot spacing or a more tightly focused beam.

As discussed in Section 2.4.2, the spatial resolution of in-operando data acquisition is constrained by the scan strategy. In this study, the part was fabricated using spot-melting strategy as described in Section 2.1. Because the surface is not continuously scanned, the distance between neighboring melt spots is approximately 250 μm distance between every spot and its neighbors in all directions. Consequently, gaps exist in the recorded signal between individual spot locations. These gaps are only affecting the signal that is being collected; however, the real spot-melts will intersect with each other due to the expenditure of the melt pool.

This melt-pool expansion occur primarily as a result of the under-selected power conditions [32]. In addition, the thermal interaction between neighboring spots can further increase the melt pool size because of remelting previously consolidated regions. This effect is particularly significant when melting occurs on previously solidified material rather than on loose powder [33]. To account for this effect, a dilation approach was implemented by measuring the visible diameter of individual melt spots on the surface of the fabricated part.

The nominal beam diameter used during melting was approximately 250 μm (FWHM) as used in Figure 3 results. However, measurement of the resulting melt pools, shown in Figure 4, indicates that the actual melt-pool diameter is significantly exceeding the nominal beam size. The average measured melt-pool diameter is 623 μm, which was used as the dilation value to compensate for the geometric expansion of the melt pools during reconstruction of the in-operando signal geometry.

Applying this dilation correction has successfully shown an overall improvement in accuracy compared with the results presented in Figure 3. This improvement supports the validity of the proposed correction approach, which could be adapted later to rely on the in-operando signal in evaluating geometry accuracy. As shown in Figure 4c, there is a significant improvement in the tooth base agreement result, where most of the values fall to under 1.5%.

Similar improvements were observed for both the chord width and the tooth tip measurements, as presented in Figure 4d and Figure 4f, respectively. The chord width depends on two boundary points located at the flanks of each tooth, which makes it sensitive to edge underestimation made by limited signal resolution. The dilation correction improved the boundary representation and reduced the measurement error relative to the final part geometry. Teeth tips are also sensitive to boundary detection that happens due to the gradual decay of signal intensity near the melt-pool edges. By compensating for this signal decay through spot dilation, the agreement at the tooth tips improved and the deviation was reduced to approximately 1 to 2% compared with higher deviations prior to the correction.

In contrast, the dilation correction results in a slight increase at the inner bore, which has minimal error increase on the agreement as shown in Figure 4e. Even though there was an increase in the error, in-operando AD still has the highest agreement with the geometry when compared to other monitoring methods. The increased deviation at the inner bore can be attributed to melting over a non-pre-melted region, reducing the melt-pool dilation. In E-PBF, melt-pool morphology is strongly governed by the underlying thermal state. Melting over consolidated or preheated material improves lateral heat conduction, promoting melt-pool spreading and uniform geometric expansion. In contrast, interaction with loosely packed or partially sintered powder, characterized by low thermal conductivity due to interparticle porosity, restricts heat flow and confines the melt pool. This will result in reduced dilation and altered solidified boundaries [33].

To assess the validity of applying a single, global dilation value across the entire gear, the diameter of each visible melt pool was measured directly from the OM image, yielding *n* = 356 individual measurements distributed across the part. The resulting distribution, shown in Figure 3b, is tightly concentrated around a mean of 623 μm with standard deviation of only 14.8 μm and a coefficient of variation of 2.4%. More than 95% of the measurements fall within ±30 μm of the mean, indicating that, for this build under the present process parameters, the melt-pool diameter is highly repeatable across the part. Propagating this dispersion through the radial dilation correction (which shifts the reconstructed radius by half the melt-pool diameter) yields an uncertainty contribution to the agreement deviation values of approximately 0.04 percentage points at the tooth tip and tooth base radii (≈17.5–20 mm) and 0.07 percentage points at the smaller inner bore radius (≈10 mm). These bounds are small compared to the reported *AD*% values, indicating that the constant-dilation assumption is justified for the gear scale and process window investigated here.

It should be noted that the dilation correction applied in this work is based on a single average melt-pool diameter of 623 μm. In practice, melt-pool dimensions during spot-melting are not spatially uniform across the fabricated part and can vary depending on local thermal history, proximity to neighboring melt spots, and local geometric conditions. Therefore, the use of a single global dilation value represents a simplification of the physical process. This approximation significantly improved the overall geometric agreement across most evaluated. As shown by the uncertainty analysis above, this assumption introduces only small (~0.04–0.07 pp) systematic uncertainty into the *AD*% values. The slightly larger residual deviation observed at the inner bore (1.36%) is therefore more likely attributable to other factors, such as the bore being melted onto a non-pre-melted region of the powder bed, rather than to spatial variability in the melt-pool diameter. Nevertheless, future work could benefit from incorporating spatially adaptive dilation models that account for local thermal conditions and melt-pool variability, particularly for thinner walls, sharp features, or builds with significant temperature gradients.

**Table 2:** Mean agreement deviation comparison between pre-melting, post-melting, and in-operando before and after spot dilation.

Comparison Points	Pre-Melting	Post-Melting	In-Operando	In-Operando Dilation
Mean AD% Tip	0.99%	0.84%	1.91%	1.51%
Mean AD% Base	4.10%	1.80%	2.43%	0.80%
Mean AD% Chord	5.65%	3.35%	2.96%	2.12%
Mean AD% Inner Bore	2.75%	2.95%	1.10%	1.36%

As illustrated in Table 2, the mean agreement deviation summarizes the overall accuracy for each technique. The results show that the in-operando signal after applying the dilation correction has excelled over post- and pre-melting in base, chord, and inner bore agreement deviations, which shows the reliability of using the in-operando signal in geometry monitoring. In contrast, in tip agreement deviation, the results have improved compared to the signal without dilation and are still considered to have a very low error of 1.51%. Signal intensity could play a big role in monitoring the melt-pool size [24], which can offer an accurate estimation of the spot dilation based on the signal, and even the surface morphology.

A comparison based on standard deviation (*σ*) indicates a clear distinction in signal stability between post-melting and pre-melting conditions. For the post-melting stage, the standard deviations remain moderate across all regions (inner circle ~0.79, base ~0.90, chord ~3.39, and tip ~0.53), suggesting a more consistent and stabilized signal after material consolidation. On the other hand, the pre-melting exhibits significantly higher variability, particularly in the base (~1.18) and chord (~4.99) regions, along with noticeable fluctuations in the inner circle (~1.93). This elevated dispersion reflects the inherent instability of the powder bed prior to melting, where lack of consolidation and surface irregularities contribute to inconsistent electron emission. Overall, the reduced standard deviation after melting confirms improved signal uniformity.

The standard deviation (*SD*) of the in-operando signal highlights the effect of dilation correction on signal stability. With dilation applied, the *SD* values for the tip, chord, bore, and base regions are ~0.79, ~1.28, ~1.29, and ~0.48, respectively, indicating relatively consistent signal behavior with moderate variability. In contrast, the corresponding values without dilation are higher, particularly in the chord (*SD* ≈ 1.75) and base (*SD* ≈ 0.66) regions, while the tip and bore exhibit *SD* ≈ 0.78 and *SD* ≈ 1.14, respectively. Overall, the reduced variability after applying dilation reflects improved signal consistency, especially in geometrically complex regions, confirming the effectiveness of the correction in stabilizing the in-operando response.

### 3.3. 3D Geometry Accuracy Compared to Actual Surface Profile

**Figure 5:**
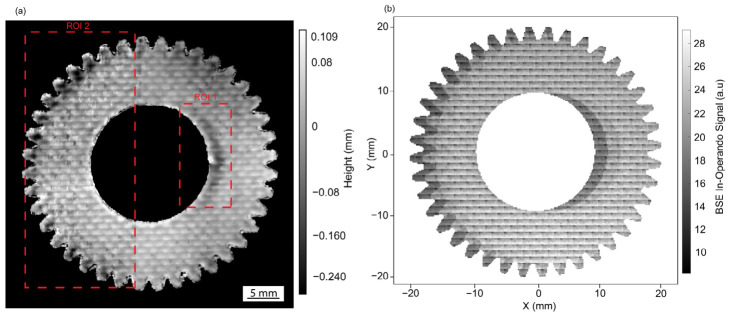
Selection of two regions of interest (ROIs) for direct comparison between the in-operando electron emission signal and the measured surface topography. (**a**) Measured surface topography of the fabricated gear, with two ROIs highlighting representative regions of distinct surface morphology: ROI 1 (centered near the inner bore) and ROI 2 (centered on the tooth region). (**b**) Spatial scatter plot of the processed in-operando electron emission data over the same field of view, where each point corresponds to one spot-melt acquisition. The two ROIs are used in the subsequent profile-by-profile correlation analysis (Figure 6, Figure 7 and Figure 8).

To assess the capabilities to capture surface topographical features of the in-operando signal of a real measured surface feature, a new comparison was made. The surface profile was measured using the KEYENCE VR-6000 profilometer (KEYENCE Corporation, Osaka, Japan) to compare it to in-melt signal deviation. As illustrated in Figure 5b, the signal intensity deviation could give a particularly good indication of the topographical features, validated by the actual surface height measurements as shown in Figure 5a. It is noticeable that the height map shares the same distribution of the signal intensity over the part surface.

In the comparison above (Figure 5), the in-operando signal showed very promising correlation to the ground truth surface measurements. The major features are presented as regions of interest (ROIs) as shown in Figure 5a. The BSE detectors signals during the melt were able to potentially capture accurate surface features with minimal additional post-processing. Both maps display distinct horizontal banding patterns across the surface, which correspond to scan strategy spot-overlapping. This specific surface feature stands out more in the BSE signal map, which reflects the sensitivity of the detectors to local beam–surface interactions and the variations in emission intensity during melting and solidification. The profilometer strengthens and confirms these variations in signal as variations in height measurements.

More distinct surface features are shown in Figure 5a, ROI 1 and 2. Those regions have a clear height difference when compared to the horizontal bands. The in-operando signal captures those features with good spatial correspondence as very clear signal variation, which can offer a very good opportunity to monitor the surface features without requiring complex surface-reconstruction approaches or even signal subtraction from opposite detectors.

This qualitative agreement between these datasets indicates that the in-operando electron signal is closely correlated with surface morphology and can effectively capture key geometric features of the fabricated structure.

**Figure 6:**
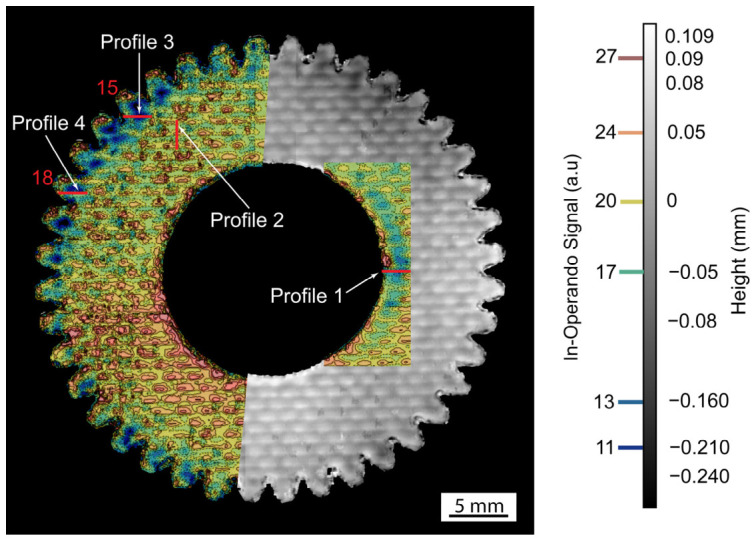
Profilometer image highlighting topographical features of the two ROIs defined in Figure 5, alongside the corresponding in-operando signal. Four spatial profile lines, two within each ROI, were defined for quantitative point-by-point comparison; the height profile measured by the profilometer along each line and the corresponding in-operando signal sampled at the same positions are analyzed in Figure 7 and Figure 8.

**Figure 7:**
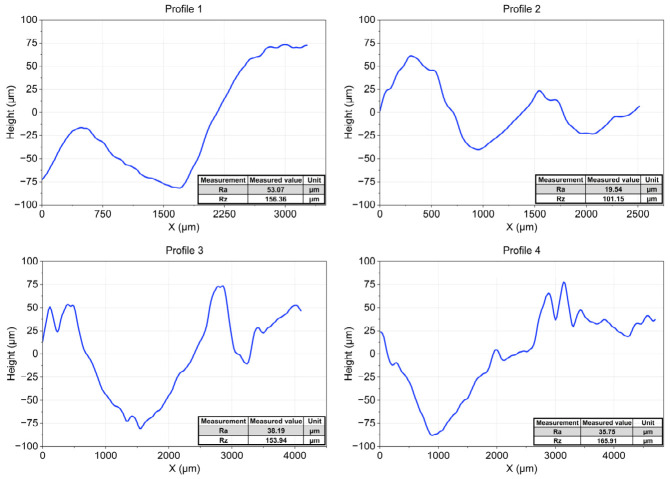
Direct surface height profiles extracted from the optical profilometer along the four pre-determined profile lines defined in Figure 6 (Profiles 1–4). For each profile, the height variation in μm is plotted as a function of position in μm. The arithmetic means roughness (Ra) and ten-point mean roughness (Rz) computed by the profilometer software (KEYENCE VR-6000 Series, Version 4.3.7.74) along each profile are reported in the inset legends. The four profiles span different physical regions of the fabricated gear surface and reveal characteristic peak-to-valley topography on the order of 100–170 μm, with Ra values ranging from 19.5 to 53.1 μm, consistent with typical as-built E-PBF Ti-6Al-4V surface roughness.

To further quantify the agreement between the in-operando signal and the measured surface topography, a statistical correlation analysis was performed between the extracted signal profiles and the corresponding profilometer height measurements along selected regions of interest as shown in Figure 6 and Figure 7.

Before the analysis, both datasets were normalized to remove differences in scale between the detectors’ signal intensity and the measured height values. Then the profiles for both in-operando and profilometer height map normalized values from specific locations as shown in Figure 6 were plotted with the same interval as in-operando signal (every 250 μm). This sampling will make it easier to compare the continued profilometer reading with disconnected data of in-operando signal intensity (Figure 5).

**Figure 8:**
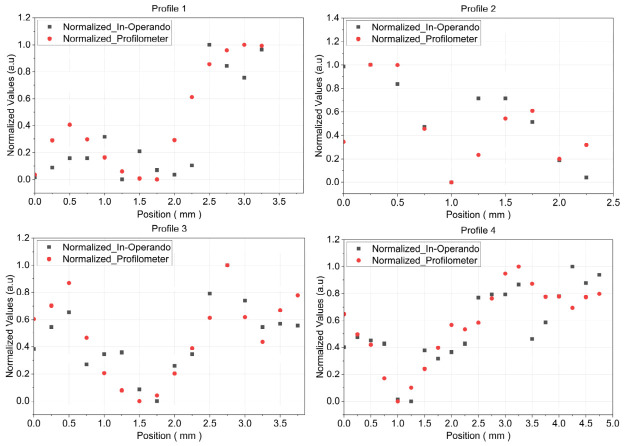
Point-by-point comparison between the in-operando electron emission signal and the profilometer-measured surface height along each of the four pre-determined profile lines defined in Figure 6 (Profiles 1–4). For each profile, the in-operando signal (red markers) and the directly measured profilometer height (black markers) are plotted at the same spatial positions to visualize the degree of agreement between signal-derived reconstruction and reference surface topography. The corresponding Pearson and Spearman correlation coefficients, *p*-values, 95% confidence intervals, and NRMSE values for these comparisons are reported in Table 3 and Table 4.

This plot (Figure 8) represents the surface topography of the determined profile after normalization. There is very high agreement in both Profile 1 and 3 at the valleys and the subsequent increases. Profile 2 shows a similar overall trend, with some local deviations. In profile 4, in-operando captures the dip and the gradual increase in surface height, but with a slight difference in amplitude. Despite these minor discrepancies, the overall trend consistency across the four profiles demonstrates that the in-operando signal is sensitive to surface morphology variations and is capable of capturing key geometric features of the fabricated part.

The similarity between the normalized in-operando electron signal and surface height profiles was evaluated using three statistical tools: the Pearson correlation coefficient, the Spearman rank correlation coefficient [34], and the root mean square error (RMSE) [35]. The Pearson correlation coefficient measures the linear relationship between the two signals and is defined as follows:

(14) $$r=\;\frac{\sum_{i=1}^{n}\left(X_{i}-\;\bar{X}\right)\cdot \;\left(Y_{i}-\;\bar{Y}\right)}{\sqrt{\sum_{i=1}^{n}{\left(X_{i}-\;\bar{X}\right)}^{2}}\cdot \;\;\sqrt{\sum_{i=1}^{n}{\left(Y_{i}-\;\bar{Y}\right)}^{2}}}$$

where *X_i_* is the normalized in-operando signal at point *i*, *Y_i_* is the normalized profilometer height at the same point, $\bar{X}$ is the mean of the normalized in-operando signal, $\bar{Y}$ is the mean of the normalized profilometer values, and *n* is the total number of the sampled points.

The Spearman rank correlation coefficient evaluates the monotonic relationship between the two datasets based on the order of the values rather than their absolute magnitudes, and is calculated as follows:

(15) $$\rho \;=\;1\;-\;\frac{6\sum d_{i}^{2}}{n(n^{2}-1)}$$

where *d_i_* is the difference between the ranks of the corresponding in-operando and profilometer values at point *i*, and n is the total number of sampled points. The ranks are assigned numbers for both datasets starting from 1 for the lowest value and increasing to reach n for the biggest value. And if there are two equal values, they will be given the average rank, so for a dataset of four ranks with two values equal to each other, their ranks will be 2.5, which is the average of the summation of the ranks’ values.

In addition, the normalized root mean square error (NRMSE) was used to quantify the deviation between the two normalized signals:

(16) $$\mathrm{RMSE}\;=\;\sqrt{\frac{1}{n}\;\sum_{i=1}^{n}{\left(X_{i}\;-{\;Y}_{i}\right)}^{2}}$$

These statistical measures allow a direct comparison between variations in the in-operando data and the measured surface height profile, enabling quantitative evaluation of how well the in-operando signal captures the surface morphology features (Table 3).

**Table 3:** Statistical correlation between the in-operando electron signal and the profilometer surface height profile.

Profile #	Pearson	Spearman Rank	NRMSE
1	0.87	0.68	0.21
2	0.67	0.71	0.28
3	0.76	0.75	0.27
4	0.80	0.77	0.18

**Table 4:** Statistical significance and 95% confidence intervals for the Pearson and Spearman correlations reported in Table 3.

Profile #	n	Pearson *p*	Pearson 95% CI	Spearman *p*	Spearman 95% CI
1	14	<0.001	[0.62, 0.96]	0.008	[0.20, 0.90]
2	10	0.032	[0.08, 0.92]	0.021	[0.10, 0.93]
3	16	<0.001	[0.42, 0.91]	<0.001	[0.38, 0.91]
4	20	<0.001	[0.56, 0.92]	<0.001	[0.48, 0.91]

As shown in Table 3 and Table 4, statistical comparisons between the in-operando electron signal and the corresponding profilometer surface height measurements (extracted directly from the profilometer reading) demonstrated moderate to strong positive agreement across all four predefined profiles. Both Pearson and Spearman analyses produced statistically significant correlations with all *p* < 0.05, and the corresponding 95% confidence intervals (using both Numby and Scipy stat) excluded zero, supporting a robust relationship between the in-operando signal and measured surface topography. Profiles 1, 3, and 4 exhibited comparatively stronger agreement, whereas Profile 2 showed moderate but significant correlation despite the smaller sample size (n = 10). NRMSE values ranged from 0.18 to 0.28, indicating generally low-to-moderate deviation between signal-derived and measured profiles.

To further evaluate whether these correlations could arise from random pairing, a non-parametric permutation test (10,000 random shuffles) was performed in Python 3.9.13 using NumPy 2.0.2 and SciPy1.13.1. The resulting null distributions were centered near zero, while the observed correlation coefficients exceeded the 95th percentile of the shuffled results for all profiles. The empirical *p*-values closely matched the analytical significance values reported in Table 4, supporting that the observed signal–topography agreement is unlikely to occur by chance. Overall, these results indicate that the in-operando electron signal captures spatial variations that correspond to measured surface height features across the sampled regions.

## 4. Conclusions

This study evaluated the capability of the in-operando electron signal to capture the geometrical and topographical characteristics of complex objects manufactured by the E-PBF process. The in-operando signal acquired during spot-melting of a Ti-6Al-4V spur gear was compared with both electron optical (ELO) images acquired before and after melting, and optical microscopy as ground truth. The following specific outcomes were obtained:

- In-operando geometry reconstruction was demonstrated directly from the electron emission signal. Spatial maps generated by correlating the emitted electron signal with beam position coordinates successfully reconstructed the global geometry of a complex spur gear during melting. Despite the inherent spatial limitation associated with the approximately 250 μm spot spacing, the reconstructed geometry captured key features including tooth tips, tooth bases, chord widths, and the inner bore with meaningful agreement to the final measured geometry
- A melt-pool-based dilation correction significantly improved geometric agreement. Direct measurement of n = 356 melt pools yielded a mean melt-pool diameter of 623 μm with a coefficient of variation of 2.4%, supporting the use of a single global dilation value under the investigated conditions. Propagating this variability introduced only approximately 0.04–0.07 percentage points of uncertainty into the agreement deviation values.
- After correction, the in-operando reconstruction achieved geometric accuracy comparable to conventional ELO imaging. The agreement deviation improved from 2.43% to 0.80% at the tooth base, from 2.96% to 2.12% at the chord width, and from 1.91% to 1.51% at the tooth tip. These corrected values became comparable to, and in some cases lower than, those obtained using pre- and post-melting electron optical imaging.
- The in-operando electron signal showed strong sensitivity to surface morphology variations. Comparison with profilometer measurements across four spatial profiles demonstrated statistically significant agreement, with Pearson correlation coefficients of 0.67–0.87 (*p* < 0.001) and Spearman correlation coefficients of 0.68–0.77 (*p* < 0.005) for most profiles. These results indicate that the spatial distribution of the signal contains quantitative information related to local surface topography.
- The practical limitations of the reconstruction approach were quantified. The minimum detectable feature size was estimated to be approximately 600–700 μm, primarily constrained by melt-pool dimensions and discrete spot acquisition. In addition, curvature-induced reconstruction bias increased at sharp features and became negligible at larger radii.
- The signal acquired during the melt fabrication of a spur gear presented in this study can be directly linked to both dimensional and surface morphology, potentially offering advantages over conventional electron-based imaging approaches that require separate imaging or additional post-processing steps.

## Data Availability

The original contributions presented in this study are included in the article. Further inquiries can be directed to the corresponding author.
